# Supplementary material for: When does humoral memory enhance infection?
Source: PLoS Comput Biol. 2023 Aug 21;19(8):e1011377. doi: 10.1371/journal.pcbi.1011377 (PMC10470880; doi:10.1371/journal.pcbi.1011377)
Supplement: S1 Text — (PDF) [file pcbi.1011377.s001.pdf]

Supplemental Text: When does humoral memory enhance infection?

Memory Dominance as a Proxy for Original Antigenic Sin

Original antigenic sin (OAS) is the situation in which the immune response remains stronger towards strains encountered earlier in life compared to strains encountered later in life. This is often seen in the context of influenza where different birth cohorts experience different strains of influenza in their youth as different influenza variants arise, compete, and disappear. Because we wanted to disentangle the nuances of some of the many potential effects of prior immunity, we focus on memory dominance instead of the more complex phenomenon of OAS. However, memory dominance can be viewed as a proxy for OAS. We consider memory dominance to occur when $A_c > A_n$ with Ac/An indicating the degree of memory dominance. Using our default parameters, if we consider the effective immune response from the antibodies to the *de novo* infection, then we can represent the change as $\Delta A_c + \Delta A_n$ where ΔA_c is the change in A_c and ΔA_n is the change in A_n both relative to baseline. We assume the effective immune response from the antibodies to the original infection is boosted by $\alpha \Delta A_c + \gamma \Delta A_n$ where $\alpha \geq 1$ and $\gamma \leq 1$. If $A_c >> A_n$ the boost to the old strain will be approximately as large or larger than the boost to the new strain leading to the OAS phenomenon.

Modeling Details

We simulate various scenarios with ADE that may cause enhancement of infection, including the effects of passive antibodies, cross-reactive antibodies, and non-antibody immune responses. We utilize a system of ordinary differential equations to compare how various initial antibody amounts affect the peak number of infected cells. A schematic of this system is shown below in Fig A and corresponds to Equations 1-8 in the main text.

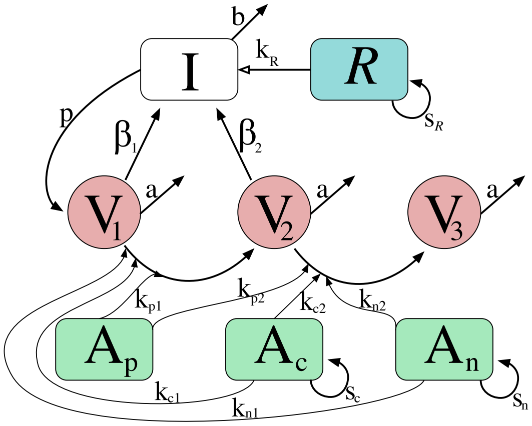

Fig A. ADE model schematic. In our model, infected cells (I) produce free virus (V_1) and can be removed via cell death or the non-antibody immune response (R). Virus moves from V_1 to V_2 to V_3 as antibody binds with only V_1 and V_2 able to infect cells. Binding can come from the *de novo* antibody response (A_n), the cross-reactive antibody response (A_c), or the passive antibody response (A_p), with A_n and A_c growing in response to the infected cells.

Similarly, we also present a schematic for the model with suppressive memory, where main text Equations 5 and 6 are replaced with Equations 9 and 10, as given in Figure B.

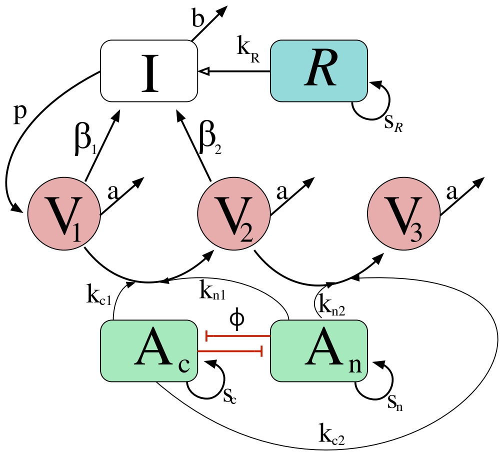

Fig B. Suppressive memory model schematic. In this model, main text equations 5 and 6 are replaced with main text Equations 9 and 10. These changes allow for direct competition, indicated by red bars, between the *de novo* and cross-reactive responses.

We give an example primary infection for both of these main text models in Figure C.

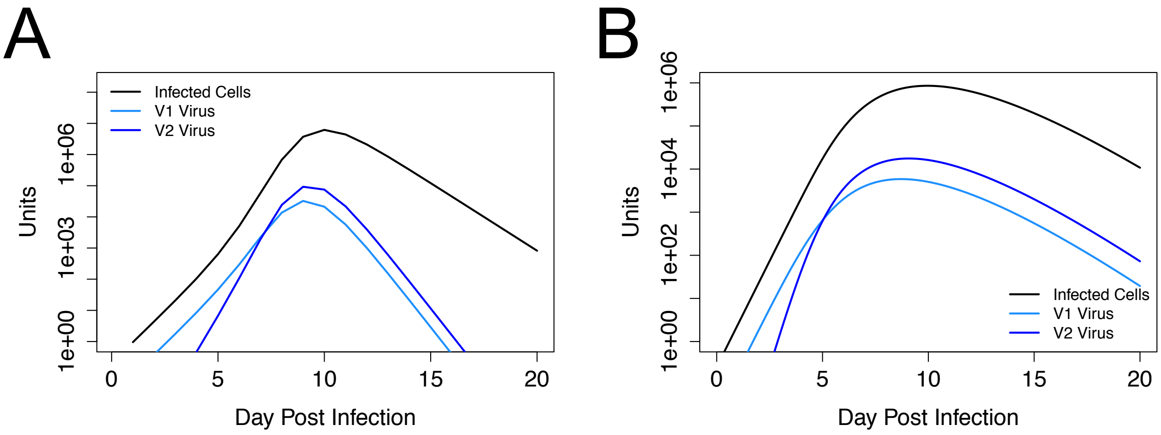

Fig C. Primary infection under both models. In Panel A, we show an example primary infection for our ADE model system, using the default parameter values. In Panel B, we show an example primary infection for the suppressive memory model ($s_R = 0$). Both have infection peaks around 10 days.

Behavior of Main Text Models

For our models we considered relevant biological regimes and matched the basic dynamics of flavivirus infections. In reality the immune response is more complex and dependent on both individual infection history along with underlying frailties, and therefore exactly matching all possible experiments and outcomes is unfeasible.

We begin by considering the simple *in vitro* case where we compare different amounts of initial antibody where partially bound virus is more infectious than free virus as shown in Fig D. While low antibody levels did not result in as many infected cells after two days, intermediate levels of antibody can lead to about 10-fold enhancement of infection, while high levels limit the infection. It should be noted that this simulation is run over a shorter time frame, as the time course of relevant *in vitro* experiments is generally quite short - no more than a couple of days.

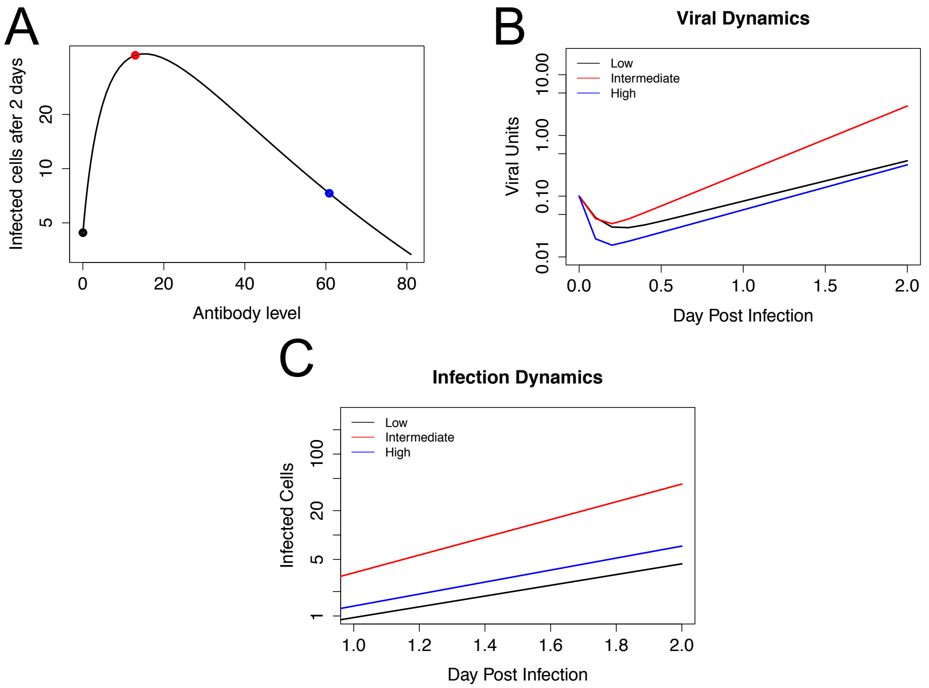

Fig D. Dynamics of infection under ADE *in vitro*. In this *in vitro* situation, where there is no growth in immune response, the phenomena of ADE is evident as the number of infected cells after 48 hours is greater at intermediate ranges of antibody. We show the differences in dynamics for three different initial antibody concentrations in Panels B and C: low but non-zero (black), intermediate (red), and high (blue).

In the circumstance where passive antibody is transferred to an individual, there is the possibility of enhancement of infection at intermediate levels of antibody transferred. However, as the individual also mounts a *de novo* response, the dynamics of infection are quite different to the previous scenario, as seen in Fig E. Here all infections peak at relatively similar timing though at different loads with intermediate antibody levels peaking at the highest amount.

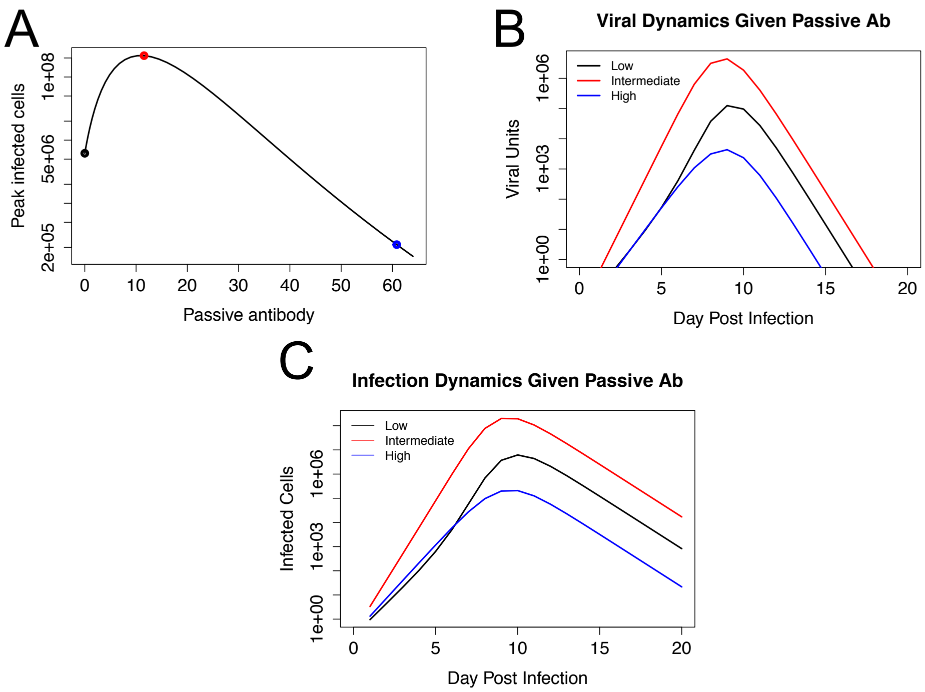

Fig E. Dynamics of infection given passive antibodies. When passive antibodies are given and a *de novo* antibody response is mounted, enhancement of infection appears at intermediate levels (red).

When cross-reactive humoral memory is introduced, dynamics change yet again. If the cross-reactive antibodies are essentially functionally the same as *de novo* antibodies, then the dynamics follow those shown in Fig F where increase in cross-reactive antibody shifts the peak timing earlier and the peak itself down. If this is the case, then there is no enhancement of infection.

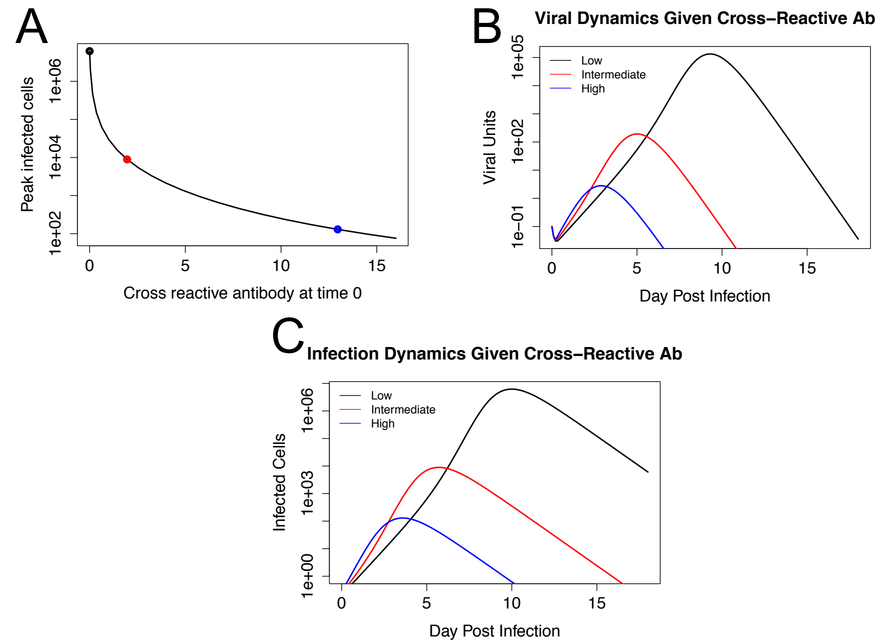

Fig F. Dynamics of infection given cross-reactive antibodies does not necessarily produce enhancement of infection in these simulations where *de novo* and cross-reactive antibodies are identical. When there are both *de novo* and cross-reactive antibodies present and growing and binding at the same rate, enhancement of infection is not seen, as shown in Panel A; rather, the greater initial cross-reactive antibody load the lower the viral load and number of infected cells as seen in Panels B-C. Here, black represents low initial cross-reactive antibody concentration, red intermediate, and blue high.

However, if cross-reactive antibodies behave differently from the *de novo* response, there may be some limited enhancement of infection as seen in G. When non-antibody immune responses are introduced into the model, the peak of infection can be lowered. However, it can cause EI-HM with viral dynamics peaking higher but clearing quicker at intermediate cross-reactive antibody levels, as seen in Fig H.

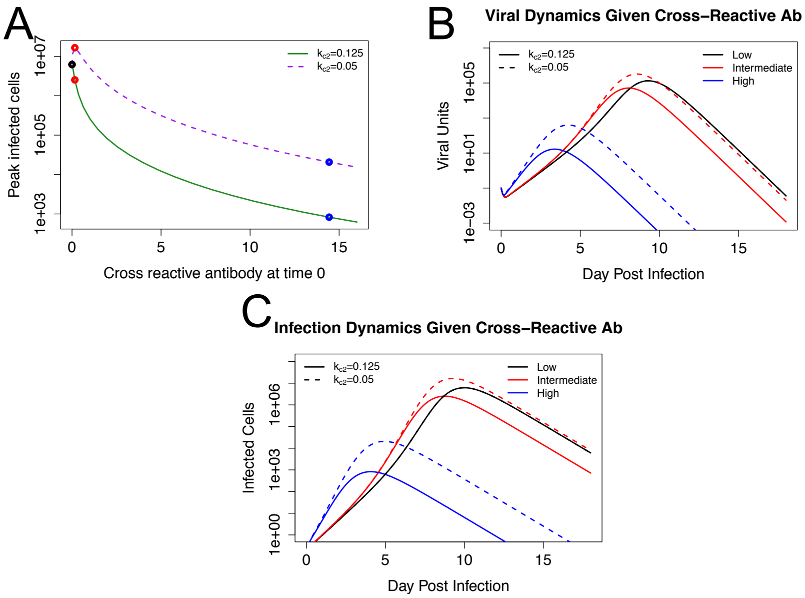

Fig G. Less neutralizing cross-reactive antibodies can sometimes cause EI-HM. If cross-reactive antibodies are approximately a fifth as neutralizing as *de novo* antibodies, some EI-HM may occur as shown in Panel A with slightly higher peak viremia and infected cell counts. While the line for the low cross-reactive antibody concentration is the same for both k_{c2} values, as seen in black in Panels B and C, they differ for the intermediate (red) and high (blue) concentrations where $k_{c2} = 0.125$ is given by the solid lines and $k_{c2} = 0.05$ by the dashed lines.

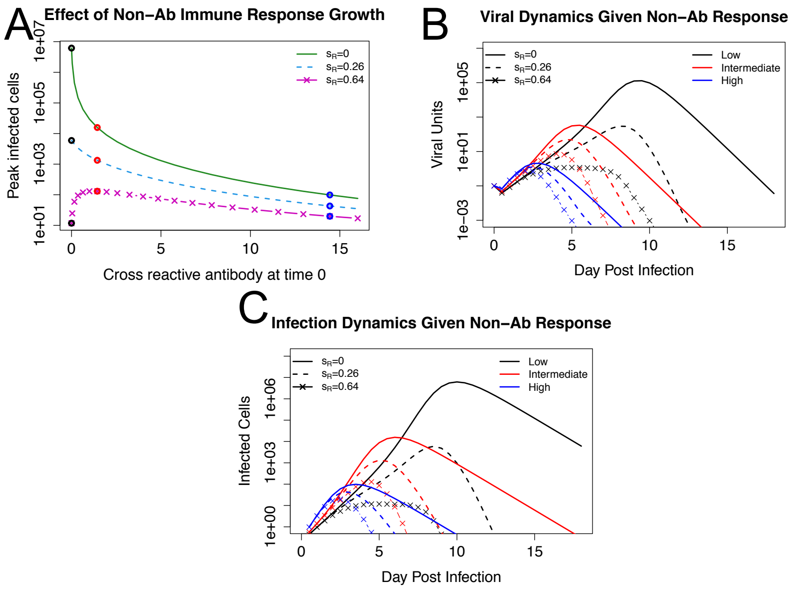

Fig H. Dynamics of infection given cross-reactive antibodies and non-antibody immune response. Depending on the rate of growth of non-antibody immune response and initial cross-reactive antibody amount, EI-HM can sometimes occur and give up to a 10-fold increase in peak infected cells. Colors denote level of initial cross-reactive antibody with black, red, and blue representing low, intermediate, and high values. Line types denote non-antibody immune response growth rates with solid, dashed, and x'd lines representing $s_R = 0$, $s_R = 0.26$, and $s_R = 0.64$ respectively.

Including suppressive memory alters the results. Here, if there are intermediate levels of cross-reactive antibodies, the infection is cleared more slowly and with a much higher peak in the absence of a non-antibody immune response. Non-antibody immune growth rate impacts the amount of infection enhancement seen, from 117 fold without it to 1.2 fold with a moderate amount of growth to no enhancement with faster growth, as seen in Fig I.

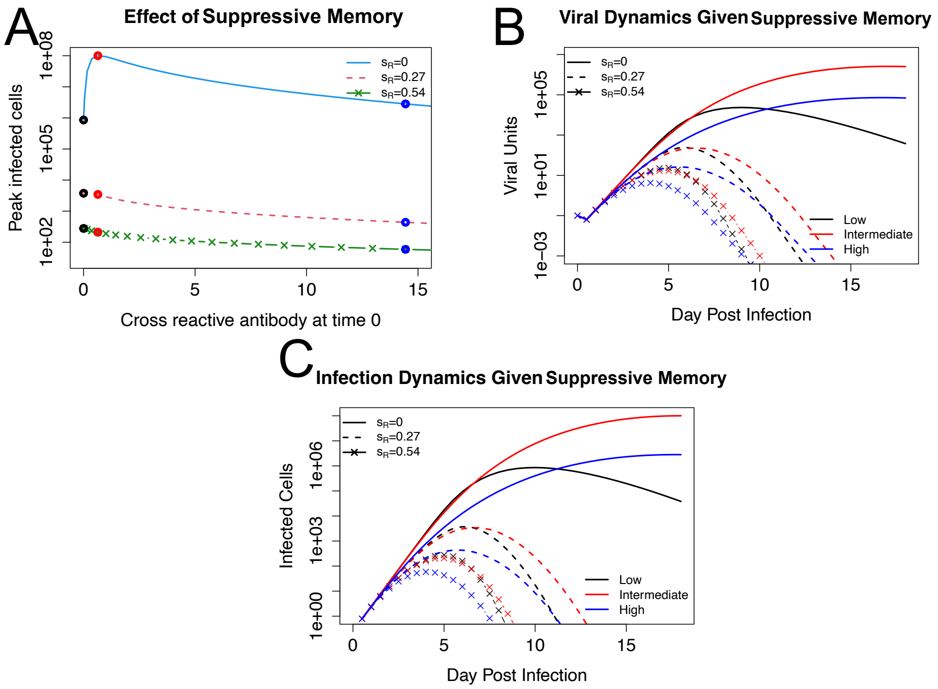

Fig I. Dynamics of infection given suppressive memory. As shown in Panel A given the scenario where there is no non-antibody immune response, EI-HM can occur with a fold change of up to 117 for $s_R = 0$. With non-antibody immune responses, however, enhancement of infection is greatly reduced with a maximum fold change of 1.2 for $s_R = 0.27$ or nonexistent for $s_R = 0.54$. In Panel B, we consider the viral dynamics for low (black), intermediate (red), and high (blue) points for each of the different growth rates where $s_R = 0$ is a solid line, $s_R = 0.27$ is dashed, and $s_R = 0.54$ is x'd. Note that for $s_R = 0$ in particular if the high point was taken at an even higher antibody level (> 25), it would in fact peak below the low point.

Other Models

We consider three changes to the basic ADE model described in § 'ADE model'. None of these changes qualitatively change our results (Figures J-N). In particular all models show ADE and EI-PA whereas EI-HM is not seen in general but is seen when 1) the cross reactive antibody is much less neutralizing (or has much higher dissociation rate) than the *de novo* antibody or 2) the non-antibody immune response is dominant in reducing peak viral load.

We also apply these changes to the suppressive memory model. Again, we see no qualitative changes (Figure O).

Change 1: B cells and plasma cells are explicitly modeled

When B cells and plasma cells are explicitly modeled, the equations for A_c and A_n in the standard ADE model are replaced by the following equations.

$$\dot{B_c} = s_c \chi(I > 1) B_c \tag{1}$$
$$\dot{P_c} = \dot{B_c} \tag{2}$$
$$\dot{A_c} = 0.05 P_C - 0.05 A_c \tag{3}$$
$$\dot{B_n} = s_n \chi(I > 1) B_n \tag{4}$$
$$\dot{P_n} = \dot{B_n} \tag{5}$$
$$\dot{A_n} = 0.05 P_n - 0.05 A_n \tag{6}$$

Here, B_c, P_c, and A_c are the B cells, plasma cells, and antibody level of the cross-reactive memory response, respectively. Analogously, B_n, P_n, and A_n represent the *de novo* immune response. Here, χ is an indicator function. For the simulations, we used $B_c(0) = P_c(0) = A_c(0)$, $B_n(0) = 0.036$, and $P_n(0) = A_n(0) = 0$. For the suppressive memory model the right hand sides of equations 1 and 4 above are multiplied by $\phi/(\phi + B_c + B_n)$ to introduce direct competition between the cross-reactive and *de novo* immune responses.

Change 2: Indicator function replaced

Step functions in the differential equations are replaced with Hill functions. Explicitly, $\chi(I > 1)$ is replaced with $I/(I + 1)$.

Change 3: Dissociation between Ab and virus is explicitly modeled

For a given virion the proportion of surface proteins bound by antibody is given by the following equations.

$$\dot{p_c} = k_{\mathrm{on,c}} A_c (1 - p_c - p_n) - k_{\mathrm{off,c}} p_c \tag{7}$$
$$\dot{p_n} = k_{\mathrm{on,n}} A_n (1 - p_c - p_n) - k_{\mathrm{off,n}} p_n \tag{8}$$

Here, p_c is the proportion of surface proteins bound by the cross reactive antibody, and p_n is the proportion bound by the de novo antibody. The association rates for the cross reactive and de novo antibody are $k_{\mathrm{on,c}}$ and $k_{\mathrm{on,n}}$ respectively. Similarly, $k_{\mathrm{off,c}}$ and $k_{\mathrm{off,n}}$ are the dissociation rates for the cross reactive and de novo antibody respectively. When solving, we discretize the above equations using N=4 binding sites. When the number of sites bound is less than or equal to Floor$(0.15 \cdot N)$, the virion is considered to be in the V_1 category. If the number of sites bound is greater than Floor$(0.5 \cdot N)$, the virion is neutralized (V_3 category). Otherwise the virion is in the V_2 category. Similar results were found at higher binding site numbers, though at the cost of computational speed. Unless otherwise stated we use $k_{\mathrm{on,\ c}} = k_{\mathrm{on,\ n}} = 0.5$/AU/day and $k_{\mathrm{off,\ c}} = k_{\mathrm{off,\ n}}$=10/day.

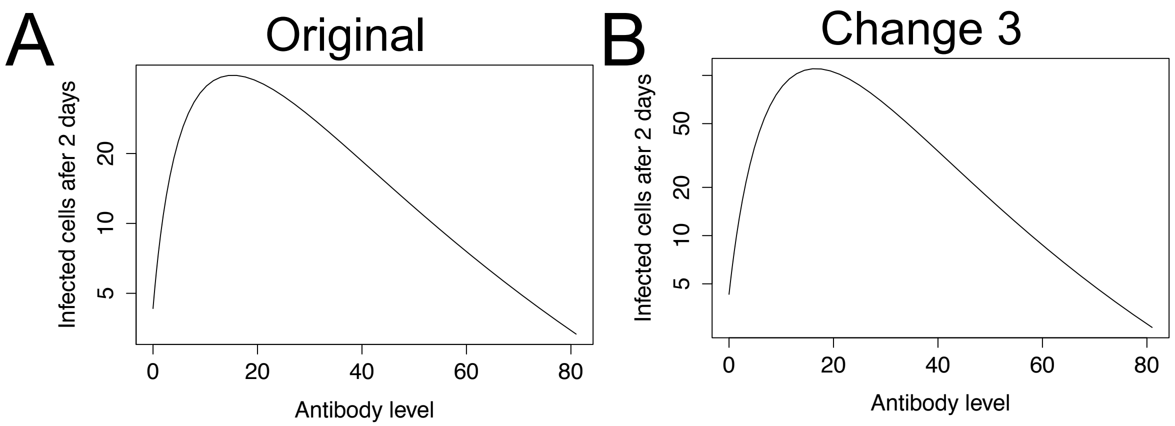

Fig J. Model changes for *in vitro* simulation. Panel A shows the original model as given in the paper while Panel B shows the model under Change 3. While this change affects the quantitative results somewhat, qualitative results remain the same with intermediate values of antibody causing enhancement of *in vitro* infection over a two day experiment. Because this is an *in vitro* experiment and there is no growth of antibodies, Change 1 and 2 are not shown as they are exactly identical to Panel A.

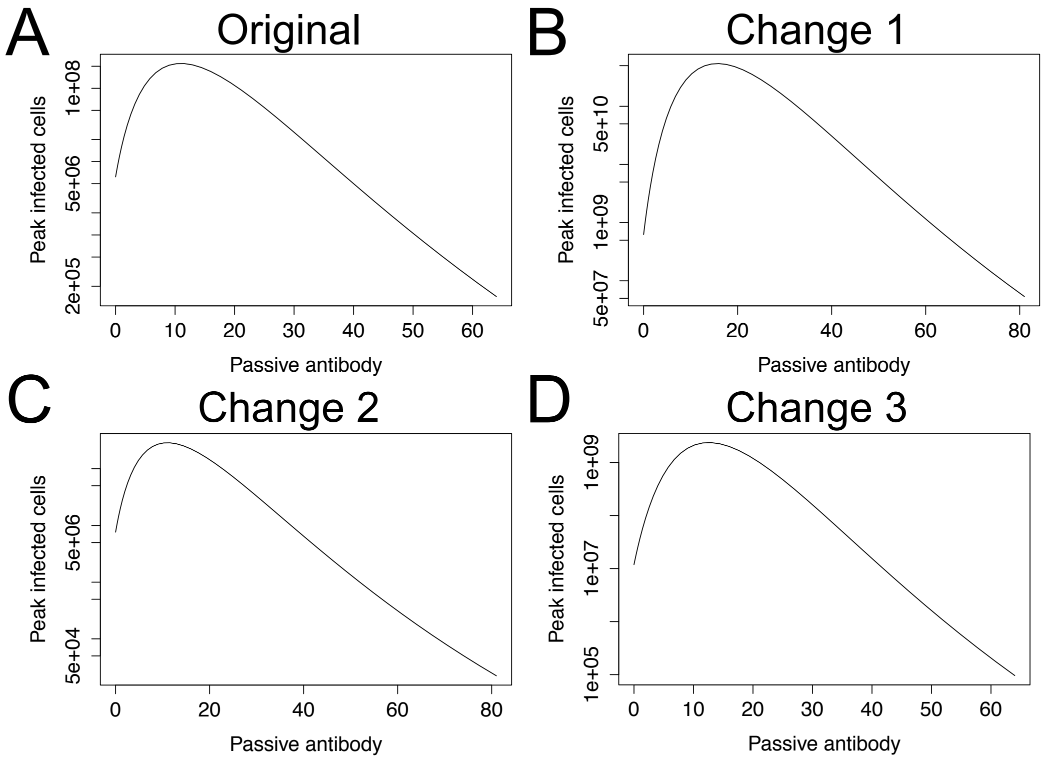

Fig K. Model changes for passive antibody simulation. Compared to the original model (Panel A), Changes 1, 2, and 3 are all qualitatively quite similar and show enhancement of infection over overlapping amounts of added passive antibody, as seen in Panels B, C, and D respectively.

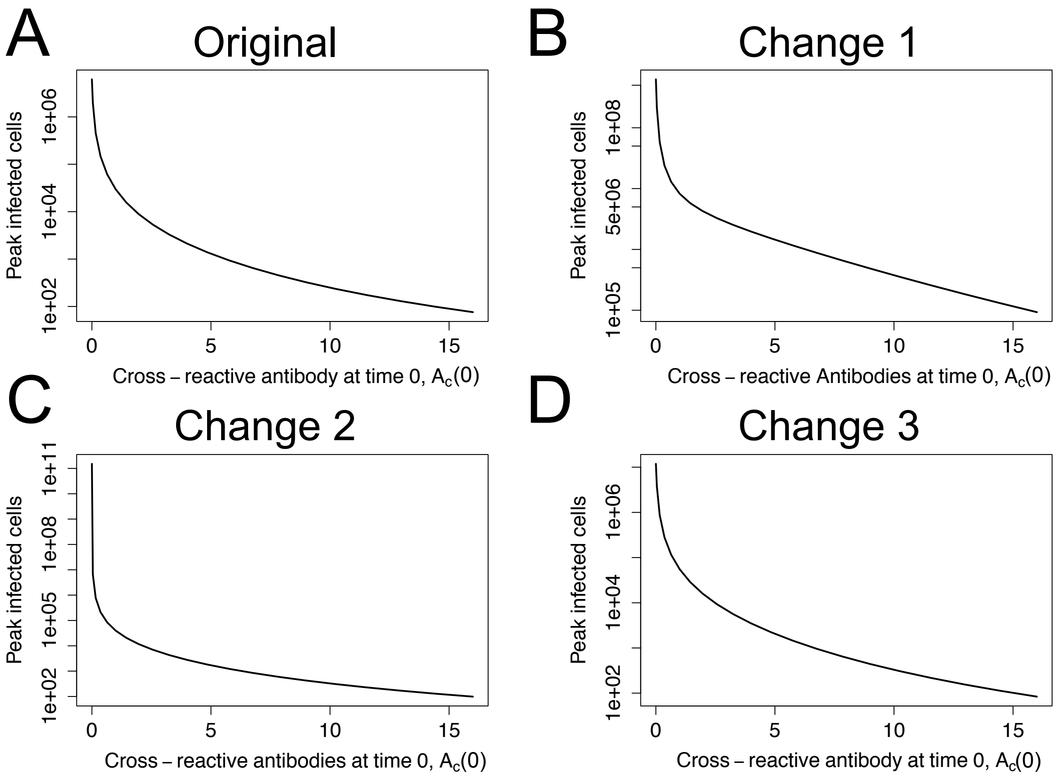

Fig L. Model changes for cross-reactive antibody simulation. For the *in vivo* simulation where cross-reactive antibodies behave the same as *de novo* antibodies, the model changes do not qualitatively alter the results; enhancement of infection is not seen regardless of initial cross-reactive antibody level.

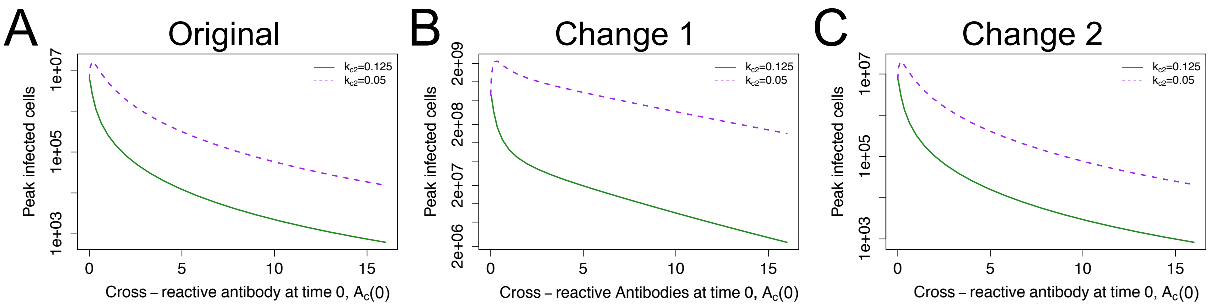

Fig M. Model changes for less neutralizing cross-reactive antibody simulation. Similar to the original model in Panel A, Changes 1 and 2, as seen in Panels B and C respectively, both show no enhancement when k_{c2} is sufficiently large and some enhancement when it is sufficiently small. Change 3 is not displayed because there is no k_{c2} parameter; however, Figure P shows a roughly analogous simulation for Change 3.

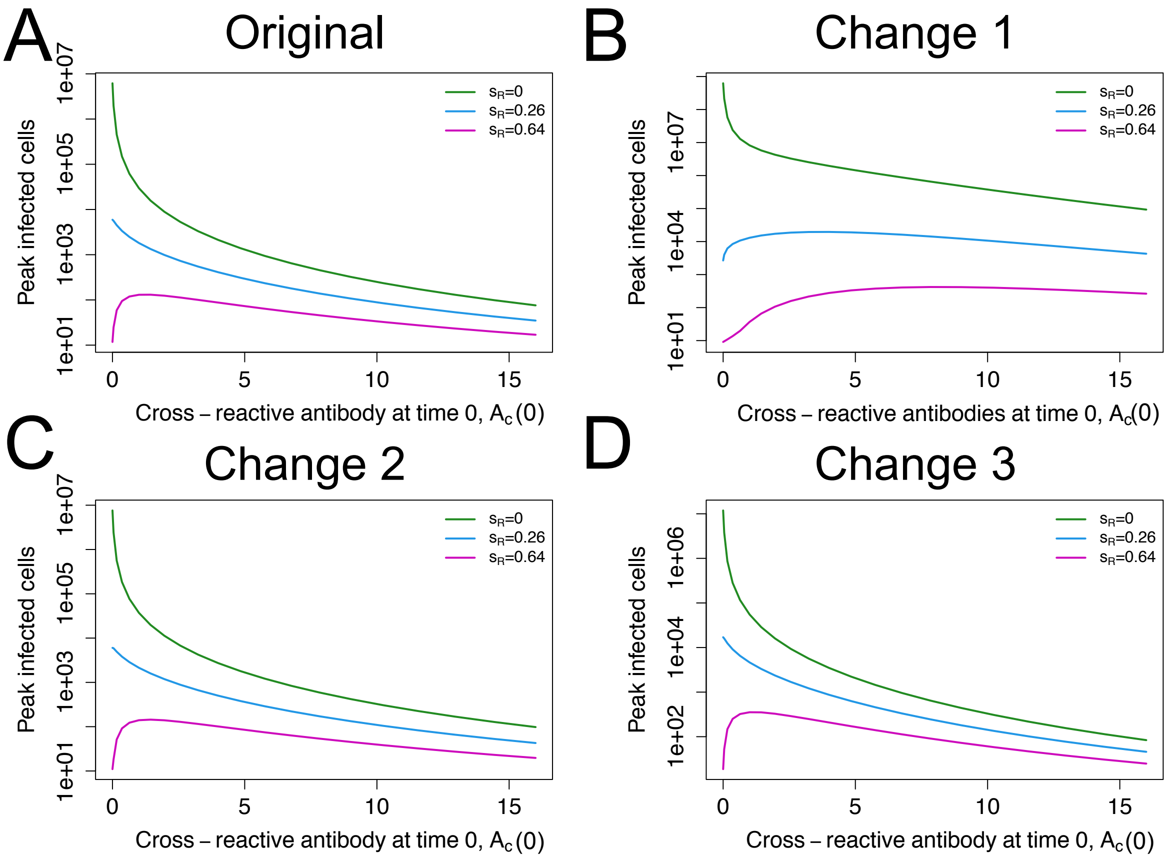

Fig N. Model changes for non-antibody immune response simulation. The model changes when non-antibody immune response is added, are qualitatively similar to the original, given in Panel A. Non-antibody immune response growth rate as given by $s_R = 0.26$ is the threshold between EI-HM and no EI-HM in the original model but not necessarily in the other models.

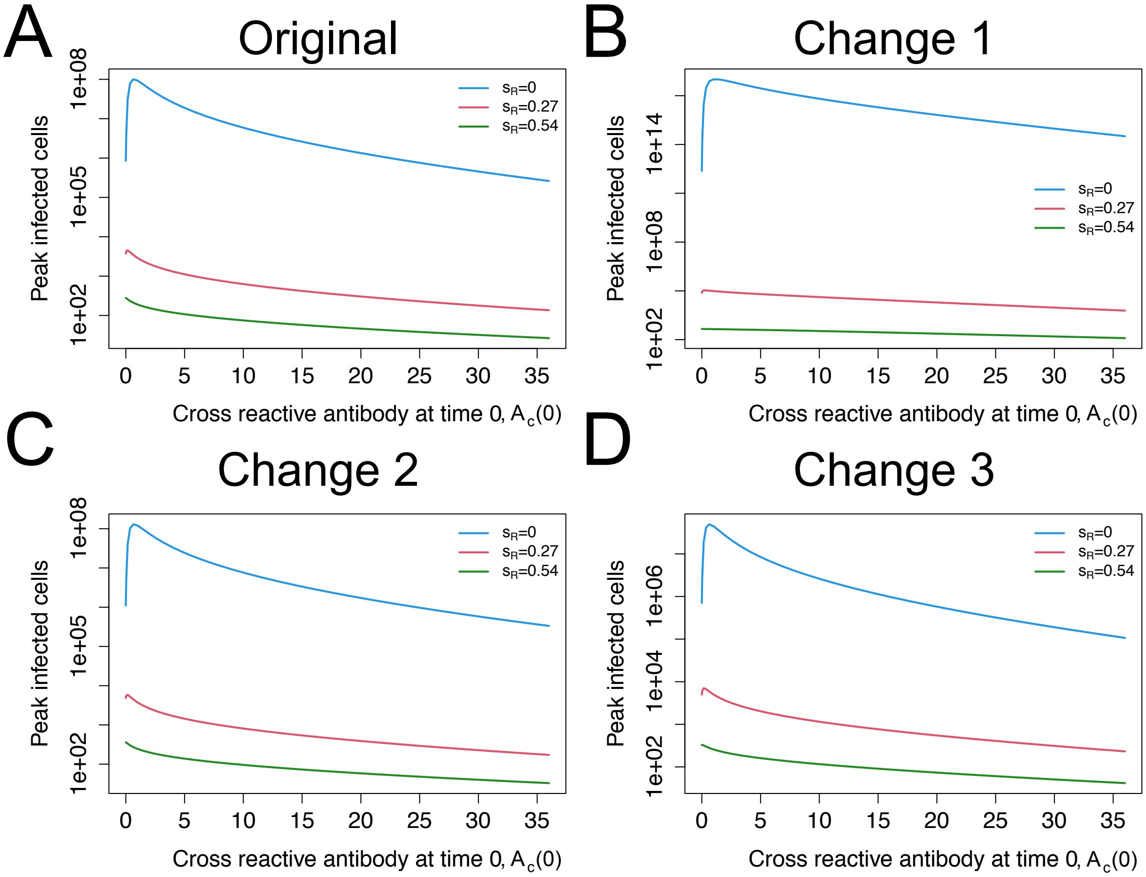

Fig O. Model changes for suppressive antibody simulation. We also applied Changes 1-3 to our suppressive memory model. The original model as well as all the changes, show enhancement when there is no non-antibody immune response as well as a reduction in enhancement as non-antibody immune response growth increases.

Cross-reactive antibody has a higher dissociation rate

For this section, we use change 3 to the basic ADE model where dissociation between antibody and virus is explicitly modeled. In these simulations, the cross reactive antibody has higher dissociation rate than the de novo antibody – either $k_{\text{off,c}} = 50/\text{day}$, $100/\text{day}$, or $150/\text{day}$ versus $k_{\text{off,n}} = 10/\text{day}$. k_{on} was held at 0.5 to make antibody units comparable across models. See [1] for experimentally measured dissociation rates for dengue antibodies. These measurements approximately span the range of 3/day to 56/day with 13/day being median. Here, values were extracted from Figure 7B in [1] using WebPlotDigitizer and converted from per second to per day. Figure P, below, shows peak infected cells for different values of $A_c(0)$.

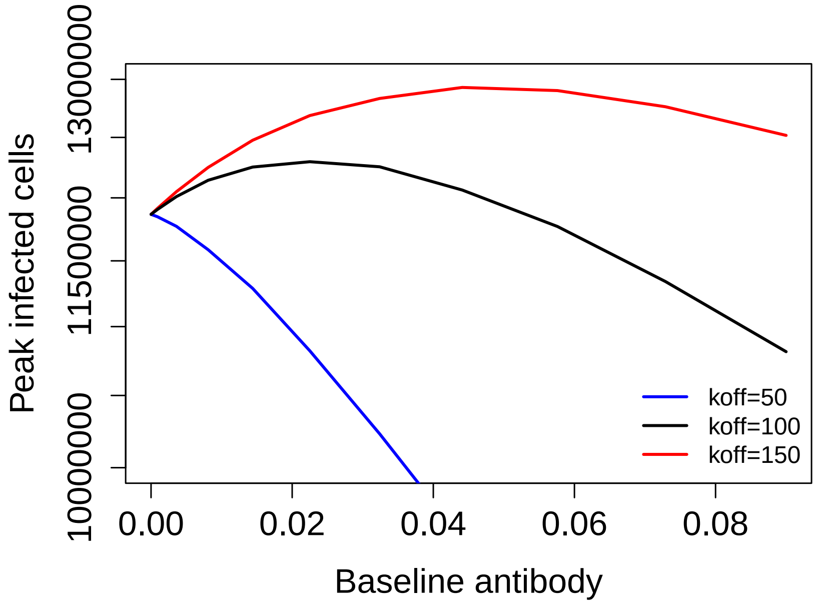

Fig P. $\mathbf{k_{off,c}} = \mathbf{100/day}$ produces some EI-HM. The figure shows simulation results from a modification to the simple ADE model such that dissociation between antibody and virus is explicitly modeled. When the cross reactive antibody has 5 times the dissociation rate of the de novo antibody ($k_{\text{off,c}} = 50$/day versus $k_{\text{off,n}} = 10$/day), there is no EI-HM. But, when the cross reactive antibody has 10 times or more the dissociation rate of the de novo antibody ($k_{\text{off,c}} = 100$/day or $k_{\text{off,c}} = 150$/day and $k_{\text{off,n}} = 10$/day), there is some enhancement of infection at low levels of baseline cross reactive antibody.

With $k_{\text{off,c}} = 50$/day, no EI-HM was observed. With $k_{\text{off,c}} = 100$/day or 150/day, there is some enhancement of infection at low levels of $A_c(0)$. But the degree of enhancement is relatively small – $k_{\text{off,c}} = 150$/day translates into maximum enhancement of only 1.1 fold compared to $A_c(0) = 0$.

Other Measures of Enhancement

In the main text models we used peak infected cell number to measure enhancement. However, there are other reasonable measures such as the area under the curve (AUC) of infected cells over time. This does not meaningfully change the results as shown in Figure Q.

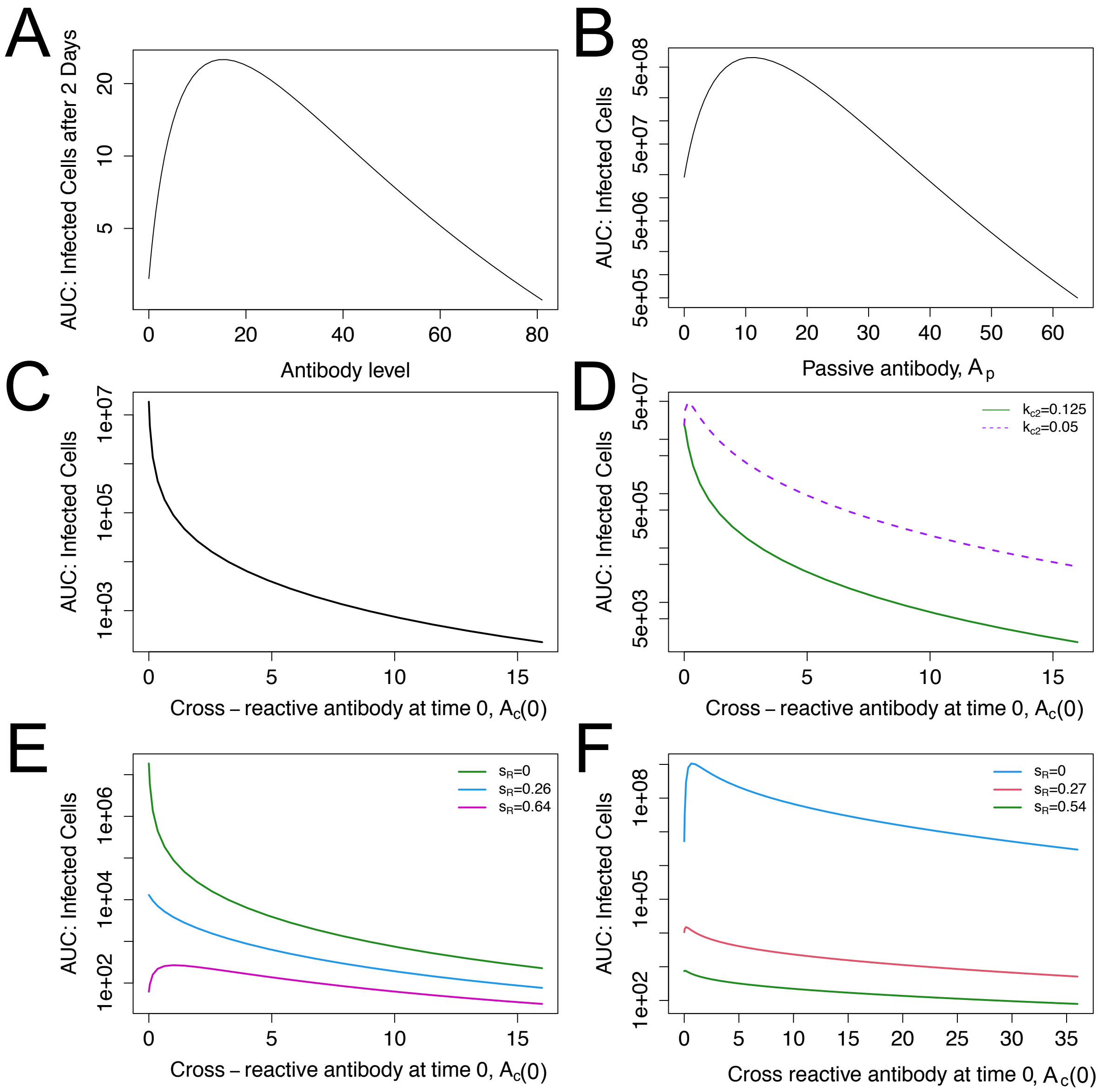

Fig Q. Alternative measure for infection load. Instead of peak infected cell number, here we use AUC as a measure of infection load. Following the main text's Figures 2-6 in order, we see that changing our measure to AUC does not meaningfully alter the results. Panels A and B show the *in vitro* and passive antibody simulations respectively. Panel C shows simulations in which cross reactive and *de novo* antibodies are similar. Panel D shows simulations in which the cross reactive antibody is 2 or 5 times less neutralizing. Panel E introduces a non-antibody immune response. Panel F shows the suppressive memory simulations.

Other adverse effects of humoral memory

We use main text Equations 1-8 with the default parameters except that there is no ADE (i.e. $\beta_1 = \beta_2 = 40$) and $s_R = 0.64$. In Figure R, we see a non-monotonic relationship between baseline antibody and final (post-infection) antibody level. Hence for antibody values above 5.8 there is a negative effect of humoral memory at baseline on humoral memory post-infection. Notably, this model

has neither ADE nor suppressive memory and hence no EI-HM.

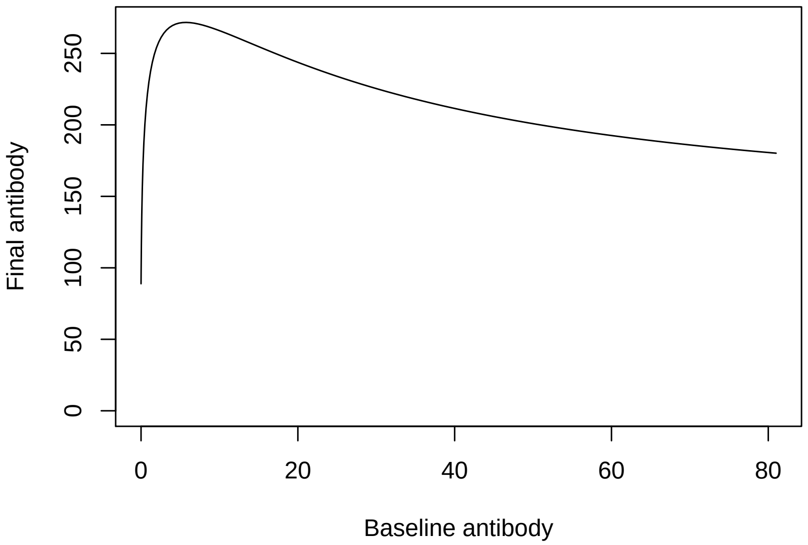

Fig R. Effect of baseline humoral memory on post-infection antibody levels. In this model, without ADE or suppressive memory, and with non-antibody immune response we see that higher levels of baseline antibody (>5.8) negatively affect final (post infection) antibody levels.

References

1. Schieffelin JS, Costin JM, Nicholson CO, Orgeron NM, Fontaine KA, Isern S, et al. Neutralizing and non-neutralizing monoclonal antibodies against dengue virus E protein derived from a naturally infected patient. Virology Journal. 2010;7:28. doi:10.1186/1743-422X-7-28.
